# Toward the Standardization of Limits to Offset and Noise in Electronic Instrument Transformers

**DOI:** 10.3390/s20144061

**Published:** 2020-07-21

**Authors:** Alessandro Mingotti, Lorenzo Peretto, Roberto Tinarelli

**Affiliations:** Department of Electrical, Electronic and Information Engineering, Guglielmo Marconi Alma Mater Studiorum, University of Bologna, Viale del Risorgimento 2, 40136 Bologna, Italy; lorenzo.peretto@unibo.it (L.P.); roberto.tinarelli3@unibo.it (R.T.)

**Keywords:** electronic instrument transformer, offset, noise, standards, accuracy, sensors

## Abstract

The scenario of instrument transformers has radically changed from the introduction of the Low-Power version, both passive and active. The latter type, typically referred to as Electronic Instrument Transformers (EITs), has no dedicated standard within the IEC 61869 series yet. To this purpose, in the authors’ opinion, it is worth understanding how the limits of typical disturbances affecting EITs should be standardized. In particular, after a brief review of the standards, the work presented a mathematical approach to determine the sources of signal disturbances influence, which affect the rms value, on the ratio error. From the results, we discussed that the emergence of disturbances generated within the EIT is a critical aspect to be studied with data of typical off-the-shelf devices. Therefore, to guarantee a correct operation of the devices, a proper standardization of the sources of disturbance should be provided.

## 1. Introduction

Instrument Transformers (ITs) are essential actors for the current and future power networks. They allow the monitoring and measurement of the electrical quantities, providing sufficient information for the grid management and control. The main standard that regulates them is the IEC 61,869 series, in which the IEC 61869-1 [1] and -6 [2] describe the general requirements for legacy inductive ITs and the more recent Low-Power Instrument Transformers (LPITs), respectively. The other documents of the series deal with specific requirements of each type of transformer (current and voltage ITs in IEC 61869-2 and -3, respectively [3,4], passive current and voltage LPITs in IEC 61869-10 and -11, respectively [5,6]), while there are no documents for the Electronic Instrument Transformers (EITs) yet. In fact, the EITs still rely on the old Standard series 60044-7 and -8 [7,8].

To this purpose, the paper aimed at contributing to the scientific world with an analysis of some peculiar aspects that affect EIT accuracy, which are worthy of standardization.

As a matter of fact, the accuracy evaluation is a critical task for all kind of electrical assets. For example, a new way of expressing uncertainty in voltage transformers (VTs) was presented by the authors of [9], while characterization and compensation techniques for their evaluation were presented by the authors of [10,11,12] (when the VT was working under non-sinusoidal condition in [10] and in a wide frequency range in [11]). An equivalent effort has been dedicated to current transformers (CTs) by the authors of [13,14,15,16,17,18]. In [13,17], for example, the authors developed new compensating techniques, while novel procedures have been developed by the authors of [14,15,16]. Even digital output ITs were well tackled by the literature [19,20]. Finally, an onsite calibration system and method were proposed by the authors of [21,22,23] for EITs, respectively, whereas their accuracy vs. harmonics was studied by the authors of [24].

However, even if the paper deals with ITs, the accuracy is an extremely important aspect for a variety of devices: cable-joints [25,26], induction motors [27], energy meters [28], electric vehicles [29], etc. This fact is mainly due to the use of the information gathered from the field, which should be reliable enough to manage and control the grid [30,31,32,33,34,35]. For example, the authors of [30,31,32,33] tackled the influence of the measurements to smart grid applications and network control, whereas the authors of [34,35] used reliable measurement collected from the ITs to run algorithms that allowed a fine control of the network.

To conclude the overview on the accuracy, it is worth mentioning that it may be affected by several influence quantities, hence, in the last years, several manuscripts have dealt with this issue. For example, accuracy vs. temperature has been discussed for EITs, CTs, and VTs by the authors of [36,37,38], respectively. Electromagnetic compatibility is another issue that affect ITs, and it has been studied by the authors of [39,40] for EITs, and VTs, respectively.

In light of the above, accuracy is the main pillar of this work. In particular, while waiting for the updated standard for EITs, what follows deals with sources of disturbances that affect the root mean square (rms), and hence the ratio error, of the quantities measured by an EITs. Such sources of disturbances, for EITs, are mainly noise and offset. Therefore, a mathematical approach was applied to find an expression that may include and quantify them in the overall accuracy evaluation of EITs. Afterward, several numerical examples were performed by implementing in the equation the limits provided by the standards to understand the weight and influence of noise and offset to the overall ratio error. Finally, dedicated comments (in light of typical datasheets taken from typical off-the-shelf devices) were given to improve and suggest ways of evaluating the uncertainty of EITs.

The remainder of the manuscript was structured as follows. Section 2 contains the detailed motivation and goals of the work. An overview of the standards was presented in Section 3 to highlight the focus of the manuscript. In Section 4, the mathematical approach to deal with the EITs uncertainty was presented, whereas in Section 5, all the numerical examples were presented together with the final comments. Last, Section 6 summarizes and concludes the overall manuscript.

## 2. Motivation and Goals

With the spread of new ITs like the LPITs, both active and passive, the need to regulate all possible aspects related to their operation increases daily. Therefore, this paper aimed to raise and deal with sources of signal distortion, introduced by the sensor itself, that may affect the rms, and hence the accuracy, of the EITs. Consequently, in the core of the text, some assumptions used to clarify that the disturbances coming from the input signal and not from the device itself were omitted and out of the scope of the work. Afterward, after an analysis of the current related standards, a general expression was obtained, evaluated, and discussed, considering two peculiar disturbances that affects the EIT: The offset and the noise.

There are two main reasons that constitute the backbone of the research. First, the EITs introduced a huge set of issues that did not affect the legacy inductive its. Second, not all of the instrumentation, adopted by the final users to acquire the measurements from the ITs, implement signal analysis techniques that extrapolate only the significant components (e.g., the DFT to extract a 50 Hz or any other component of interest for a specific application). This second aspect is critical because the “manufacturers’ secrets,” or, in other words, the technologies implemented for each task, are not public, hence it is very difficult to state that a particular task is performed by everyone in the same way. Furthermore, the solving of the issues related to EITs accuracy cannot be demanded by a third party but it should be tackled by specific standards. This aspect is critical to avoid the spread of custom solutions that go in the opposite direction of a harmonized EITs scenario.

## 3. Standards Overview

This section is dedicated to the understanding and review of what is currently prescribed by the standards related to ITs.

As previously mentioned, the standard of interest is the IEC 61869-6 [1], which provides the general requirements for the LPITs and replaces part of the old IEC 60044-7 and -8. In fact, the following documents of the series IEC 61869-10 and -11 [5,6] are dedicated to Low-Power Current Transformers (LPCTs) and Low-Power Voltage Transformers (LPVTs), respectively, while the documents related to EITs (IEC 61869-7 and -8) are currently being written by the Technical Committee TC-38.

**Figure 1:**
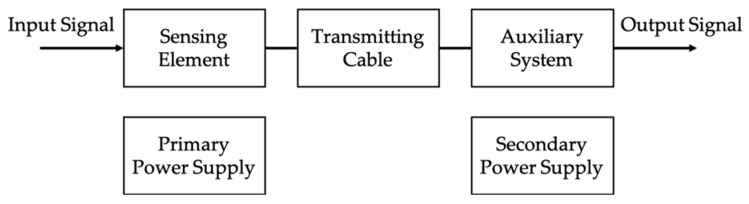
Generic block diagram of a single-phase Low-Power Instrument Transformers (LPIT) (from [2]).

Standard [2] is valid for both active and passive LPITs, which have a generic block diagram like the one depicted in Figure 1 (taken from [2]). From the picture, it is clear that the output signal, either analog or digital, is not manipulated by any digital processing technique.

Furthermore, the output signal is defined as:

(1) $$y_{S}\left(t\right)=Y_{S}\sqrt{2}\sin\left(2\pi ft+\varphi_{S}\right)+Y_{sdc}+y_{Sres}\left(t\right)$$

where f is the fundamental frequency, t the time variable, and $\varphi_{S}$ is the secondary phase. $Y_{sdc}$ is the secondary direct signal, and $y_{Sres}\left(t\right)$ the secondary residual signal including harmonic and subharmonic components. Finally, $Y_{S}$ is the rms value of the secondary converter output when both $Y_{sdc}$ and $y_{Sres}\left(t\right)$ are equal to zero.

Note that (1) is valid for analog signals and for digital ones when t is replaced with n (samples variable). Furthermore, it is clear from (1) that the output signal of an LPIT may include sources of distortions, which alter the pure sinusoidal signal, like the direct component, harmonic, and subharmonic content. This latter aspect becomes interesting when looking at the definition of the ratio error ε in (2):

(2) $$\varepsilon =\frac{K_{r}Y_{S}-Y_{p}}{Y_{p}}\times 100,$$

where $K_{r}$ is the rated transformation ratio and $Y_{p}$ is the rms of the fundamental component of the input signal. Hence, by adopting $Y_{S}$, it follows that the secondary term used to compute ε only consists of a fundamental component, cleaned from all kind of disturbances, either introduced by the sensor or by the primary signal.

Any indication of which should be the limits for direct component ($Y_{sdc}$) and for the signal-to-noise ratio (SNR) is missing from Reference [2]. As for the latter disturbance, the authors of [2] only specified that the SNR should be provided by the manufacturers in the datasheets of the devices. Furthermore, no indication on how and if such disturbance components may affect the accuracy the LPITs was given. In fact, the direct component and the noise directly affected the rms value of the measured quantity, hence they affected all quantities that were computed starting from a rms information (for example the apparent power).

Overall, the general comment is that a document like [2], which provided requirements for all kind of LPITs, should contain the abovementioned information. In support to this statement, Reference [2] also covered EITs and, as active devices, was more likely to introduce issues like noise and direct components superimposed to their output signal. Furthermore, Reference [2] did not provide a real evaluation of the LPITs’ accuracy, even in the ideal sinusoidal condition. In fact, from how ε was defined, its computation used only “cleaned” parameters which did not consider any disturbances that could be introduced by the sensor itself. This may lead, as detailed in what follows, to an underestimation of the uncertainty, especially when the input quantity is rather lower than the rated value.

## 4. Proposed Approach

To deal with the issue presented in the previous section, the authors started from the rms and ratio error expressions to obtain a compact relation that links the accuracy of an LPIT to the signal disturbances, caused by the sensors, that affect the rms quantities.

Let us start from the definition of the rms value of a generic quantity X at the output of an LPIT:

(3) $$X=\sqrt{X_{0}^{2}+X_{N}^{2}+X_{1}^{2}+X_{h}^{2}};$$

which has been written highlighting four main terms: The direct $X_{0}$, the rated value at fundamental frequency $X_{1}$, the one that summarize noise $X_{N}$, and all other components included in the signal, summarized with $X_{h}$. From here on out, the term $X_{h}$ was neglected, assuming that the primary signal did not include any harmonic, subharmonic, etc., and that the device under test was linear. Hence, all possible sources of disturbance were limited to offset and direct components and attributed to the sensor. Such an assumption is fundamental to focus on the offset and noise introduced by the EITs. Moreover, from a numerical point of view, the effect of nonlinearity is usually much lower than those of the considered disturbances. Therefore, (3) can be written has:

(4) $$X=\sqrt{X_{0N}^{2}+X_{1}^{2}},$$

if the direct and the noise components are merged into $X_{0N}^{2}$.

Afterwards, it is worth defining the ratio o between those two sources of distortion and the fundamental signal at rated value, assuming, as it is usually done, that $X_{0N}$ is independent of the input signal:

(5) $$o=\frac{X_{0N}}{X_{1}}.$$

Hence, after some basic manipulation, (4) becomes:

(6) $$X=X_{1}\sqrt{1+o^{2}},$$

where (6) holds for measurements performed at the rated value $X_{1}$. To generalize (6), for whatever input quantity value, the variable $X_{t}$ is defined as:

(7) $$X_{t}=pX_{1},$$

where p represents a factor that scales the rated value to the selected testing value (*p* typically ranges between 0.01 and 2). This means that $X_{t}$ is the rms value of a sinusoidal component at rated frequency.

Hence, (4) can be written as:

(8) $$X^{\prime }=\sqrt{X_{0N}^{2}+{\left(pX_{1}\right)}^{2}}=\sqrt{{\left(\frac{oX_{t}}{p}\right)}^{2}+{\left(X_{t}\right)}^{2}}=X_{t}\sqrt{{\left(\frac{o}{p}\right)}^{2}+1};$$

which leads to find the ratio between the measured value $X^{\prime }$ and the “real” one $X_{t}$:

(9) $$\Delta_{x}=\frac{X^{\prime }}{X_{t}}=\sqrt{{\left(\frac{o}{p}\right)}^{2}+1}.$$

The quantity $\Delta_{x}$ represents the contribution of the direct current (offset) and the noise components to the measured quantity. In other words, it expresses a ratio that highlights the effect of the nonlinearities introduced by the sensor. A relation similar to (9) can be obtained from the definition of ε in (2):

(10) $$\Delta_{\varepsilon }=\frac{K_{r}Y_{S}}{Y_{p}}=\frac{\varepsilon }{100}+1,$$

which reflects the ratio between the primary and secondary quantity (with the former scaled to the primary side).

At this point, if (9) and (10) are merged, the relative value o obtained from a specific ε is:

(11) $$o=\pm p\sqrt{\frac{\varepsilon^{2}}{10^{4}}+\frac{\varepsilon }{50}}.$$

In other words, (11) is the expression that assumes that the ε of a LPIT is all due to the contribution of noise and direct components, represented by o (hence, the LPIT is working in ideal conditions except for those two contributions). To obtain o in absolute value, it is sufficient to multiply it for $X_{1}$. Of course, as written in Section 3, the current standards do not take into account offset and noise in the evaluation of the ratio error but, in actual conditions, the above nonidealities lead the rms of the LPIT output to differ from the one obtained from ideal and rated conditions, thus turning into a “ratio error.”

The final step of the mathematical development consists of improving (11) to make it significant in practical applications. In fact, in its current form, it assumes that ε is all due to noise and direct components. Therefore, it is reasonable to introduce a coefficient k that specifies a portion of ε that may be caused by those two sources of disturbances. The use of a coefficient like k is already a common practice by the authors of [6] where, for example, the test vs. electric field is valid when ε varies no more than ε/5 between the test with and without the presence of electric field.

Consequently, (11) can be rewritten as:

(12) $$o=\pm p\sqrt{\frac{\varepsilon^{2}}{k^{2}10^{4}}+\frac{\varepsilon }{50k}},$$

to include the k coefficient. From the authors experience, it is possible to state that a range of values that may be attributed to k is 3–5. In other words, with (12), it is possible to compute the maximum value of o that makes its effect almost negligible with respect to the ratio error allowed by the accuracy class of a given LPIT.

## 5. Numerical Examples

### 5.1. Percentage Evaluation of o

In this section, the expression (12) obtained in Section 4 was applied to the testing values reported by the authors of [5,6]. Of course, considering that the standards for EITs are not available yet, it was assumed here that what was prescribed for passive LPITs could be extended for EITs (which is reasonable in light of their “low-power” common feature).

The information needed to compute numerical examples starting from (12) are mainly: (i) The ratio error of the device, (ii) its rated output, and (iii) the standardized input signal amplitude. Of course, another required information is k. However, it is mainly selected by experience and common sense. To this purpose, in all the numerical examples that follow, k=3 was adopted and implemented.

Table 1 lists the rated outputs suggested by the authors of [5,6] for LPCTs, and LPVTs, respectively. Note that, from the table, it is already possible to state that the current devices were more prone to suffer from signal disturbances, considering the low amplitude of the standard outputs.

Turning to the testing signals, LPCTs and LPVTs featured different values, which are collected in Table 2, including both measuring and protective categories of devices. In the table, $V_{n}$ and $I_{n}$ refer to the rated voltage and current of the device, respectively. The values in the table hold for all the accuracy classes defined for LPCTs and LPVTs by the standards.

With the three required inputs, it is possible to apply (12), obtaining the relative value o for every combination of accuracy class (AC) and testing level specified by the standards. For a better understating, see Table 3, Table 4, Table 5 and Table 6, which contain all the combinations and related results. For the sake of comprehension, tables should be read as, e.g., “*for the accuracy class 0.2 and amplitude of the input signal of* $0.2I_{n}$*, the ratio error* ε *defined by the standard is 0.35% and the computed parameter* o *is 0.97%*”.

In the four tables, o is presented in percentage to be understandable at a glance. Furthermore, being numerical and not experimental examples obtained from ITs, results are presented with two significant digits.

Focusing on Table 3, it is possible to note how the limit imposed to o becomes stricter has the testing current decreases. In fact, as an example, for a typical 0.5 AC, the maximum contribution of noise and direct components compared to the fundamental was 5.77% when the testing current was the rated, while it decreased to 0.50% when the test was performed at 0.05$I_{n}$.

A comment from Table 5 is that, considering that the measuring LPVTs were tested at high voltage levels (±20% of the rated) compared to the LPCTs, the limits fixed by o were less stringent and never lower than 2%. The same cannot be stated for protective LPVTs because the low testing levels resulted in a very limited allowed presence of noise and direct components.

### 5.2. Absolute Evaluation of o

To better understand the figures collected from the numerical examples in the tables of the previous subsection, the following graphs present the related absolute values. In particular, as introduced before, the output values listed in Table 1 were used to obtain o in absolute terms.

**Figure 2:**
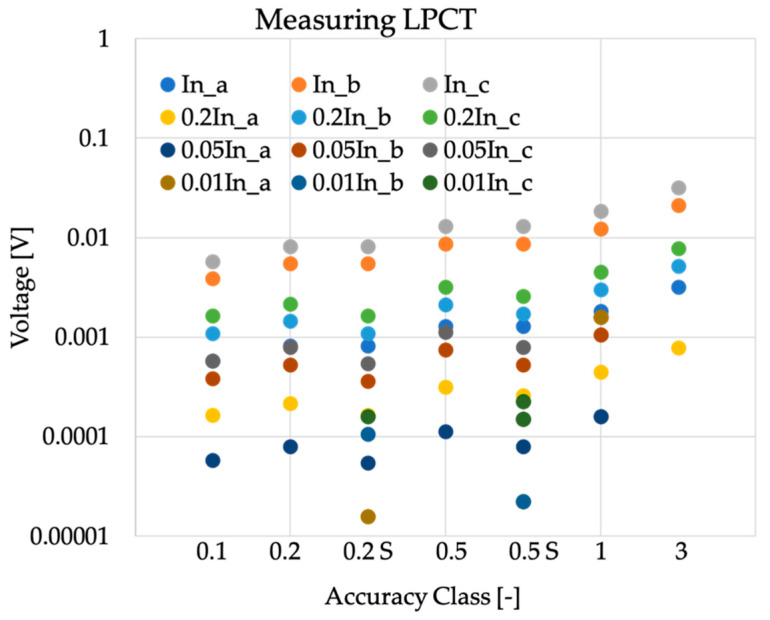
Absolute values of o computed from the rated outputs of Table 1 for measuring purpose current transformers.

In Figure 2 the absolute values of o were graphed starting from the rated outputs listed in Table 1 (referred to in the graphs as (*a*), (*b*), and (*c*)). The graph contains all the values associated with Table 3 by adopting a logarithmic scale, which better highlights the range of values assumed by o. It is clearly emphasized from the graph that, regardless from the accuracy class observed, the maximum allowed contribution of noise and direct component was of few millivolts (remember that we used a k=3). Such limits are more stringent as the testing current decreases, and overall, they are very difficult to maintain compliance with in practical situations.

**Figure 3:**
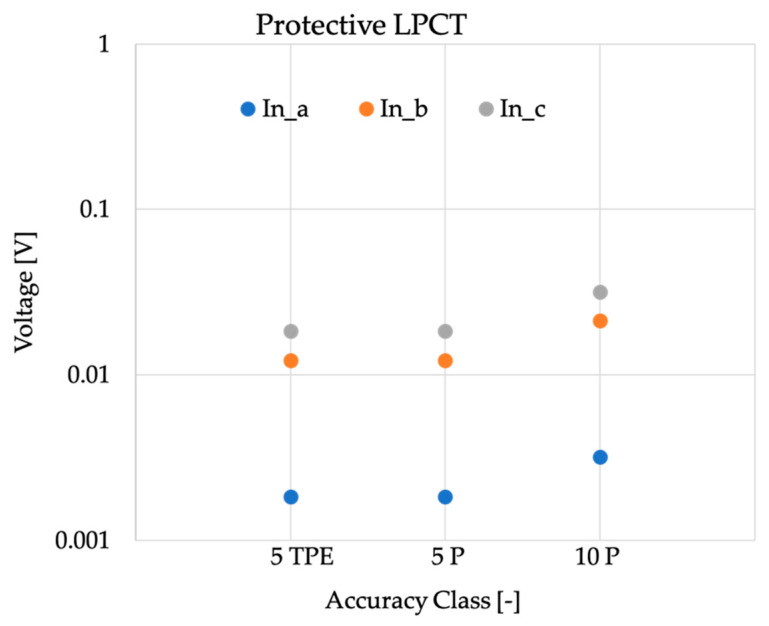
Absolute values of o computed from the rated outputs of Table 1 for protective purpose current transformers.

Things improved a little when dealing with protective devices. In fact, from Figure 3, it is clear that the higher ε values allowed from Table 4 resulted in less stringent limits on o. Therefore, the average allowed contribution of noise and direct component was of tens of millivolts, with the exception of the rated output (*a*) (22.5 mV), which only allowed a maximum of 3 mV contribution.

**Figure 4:**
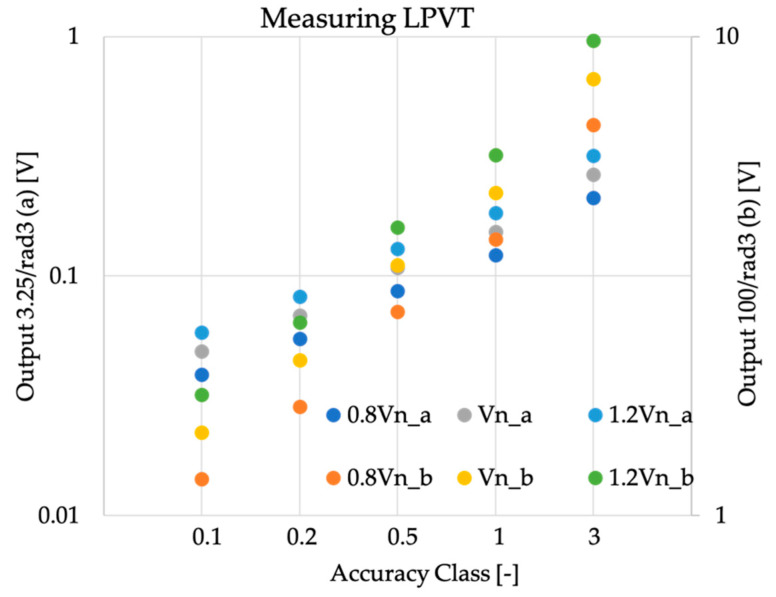
Absolute values of o computed from the rated outputs of Table 1 for measuring purpose voltage transformers.

Turning to voltage transformers, numbers drastically changed due to the adopted testing levels and fixed rated outputs. In fact, absolute values obtained for the rated output $100/\sqrt{3}$ varied from 1 V to 10 V, hence far greater than a typical instrument noise level of direct component. However, even for the other rated output, $3.25/\sqrt{3}$, the limits imposed by o ranged from 50 V to 300 mV, which are reasonable values easily met by off-the-shelf instrumentation. The comments can be confirmed looking at Figure 4.

**Figure 5:**
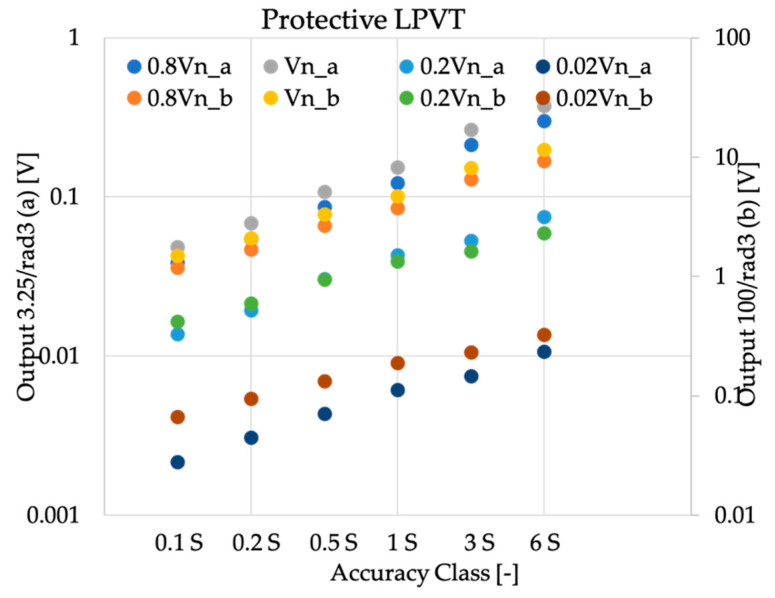
Absolute values of o computed from the rated outputs of Table 1 for protective purpose voltage transformers.

Finally, the situation slightly degraded for protective purpose voltage transformers, as it can be seen in Figure 5. The fact is simply due to the variation of the testing levels and ratio error limits (as presented in Table 6). However, even for the smallest $0.01V_{n}$ voltage level, the allowed noise and direct components contribution was of few millivolts.

### 5.3. Comments and Proposals

In addition to the set of presented results, the study was completed and commented on, considering the information gathered from some off-the-shelf EITs and looking for specifications related to offset and direct components thresholds. To this purpose, 20 EITs (current/voltage, of various technology, and for different voltage level applications) from different manufacturers were found and analyzed. We observe that: (i) 12 manufacturers did not provide any indication on the offset or the noise introduced by the EIT; (ii) 6 manufacturers provided only the offset threshold of their EITs, and (iii) 2 manufacturers provided both information on noise and direct components. For the second and third groups of devices, the thresholds features are collected in Table 7. Note that the names of the manufacturers were omitted (replacing them with letters) for the sake of privacy and considering that the aim was not to point out who is providing what, but just to obtain a clear idea of which kind of information is available from the off-the-shelf EITs. In Table 7, in addition to the abovementioned thresholds, the main characteristics of the EITs were presented, together with a column which describes whether or not the EIT was compliant with the limits obtained in Section 5.1. To compute such values, we assumed that the EITs were for measuring purposes, considering that this information was not specified by the manufacturers.

Therefore, in light of Table 7 and of the set of results presented in the previous subsections, it can be concluded that:

- Only the 10% of the manufacturers provided the information on both noise and direct components values. Instead, 40% of them, at least, provided information on one of the two features.
- Even for non-experts, it is simple to conclude that the limits obtained with the proposed approach are not impossible to be met, but quite severe. In particular, as expected, the limits were less stringent for the measuring voltage sensors. Therefore, the devices listed in Table 7 were all compliant with the limits except for device *C*. Turning to current devices, it was confirmed that the limits were quite stringent, and off-the-shelf devices, as those in Table 7, had difficulties in fulfilling the accuracy requirements. Finally, it has to be highlighted that the devices in the table that are compliant with the limits are the most expensive compared to the others.
- The previous point raises the need of standardization of those aspects, including a review and improvement of [2] to better deal with EITs.
- Last, but perhaps the most important comment, is that further efforts could be spent on improving the definition of ε to include those practical aspects and real components that affects realistic signals. In fact, at the current status the computation of ε involves the use of only fundamental components, “cleaned” from all kind of disturbances and real-world components. Therefore, a harmonization and clarification from the standards is required to prevent the accuracy from becoming a measurement laboratory aspect and not a broader consolidated knowledge.

## 6. Conclusions

This paper aimed to raise a particular issue affecting electronic instrument transformers. In fact, considering their working principle, they are more affected by sources of disturbance than other instrument transformers. Such disturbances act on the rms quantities, and hence the ratio error, measured by the transformers.

Therefore, considering that proper standards do not deal with such issues yet, the paper presented a mathematical expression which emphasized the contribution of noise and direct components to the overall ratio error index. Afterwards the expression was applied on practical numerical examples for evaluation, on absolute terms, which limits the manufacturers need to comply.

The results showed that the limits obtained with the rigorous mathematical expression were, in most cases, severe. To prove that, such limits were compared with those gathered from typical off-the-shelf devices (both current and voltage transformers). The comparison clearly confirmed that commercial electronic current transformers have more difficulties in meeting the limits than the voltage counterparts. In particular, only expensive equipment is capable of fulfilling the fixed limits.

Therefore, the authors believe that there is a strong need for standardization in this field, due to the significant relevance of the source of disturbance on the overall accuracy of an electronic instrument transformer.

## Figures and Tables

**Table 1:** Rated outputs, taken from IEC 61869-10 and -11, for LPCTs and LPVTs, respectively.

LPVT [V]	LPCT [mV]
$3.25/\sqrt{3}$	22.5
$100/\sqrt{3}$	150
	225

**Table 2:** Testing levels, taken from IEC 61869-10, 11, for LPCTs and LPVTs, respectively.

*Type*	*Testing Levels*			
*Measuring LPCT*	$0.01I_{n}$	$0.05I_{n}$	$0.2I_{n}$	$I_{n}$
*Protective LPCT*	-	-	-	$I_{n}$
*Measuring LPVT*	$0.8V_{n}$	$V_{n}$	$1.2V_{n}$	-
*Protective LPVT*	$0.02V_{n}$	$0.2V_{n}$	$0.8V_{n}$	$V_{n}$

**Table 3:** Results for measuring purpose LPCTs.

*AC*	$0.01I_{n}$		$0.05I_{n}$		$0.2I_{n}$		$I_{n}$	
	ε **[%]**	o **[%]**	ε **[%]**	o **[%]**	ε **[%]**	o **[%]**	ε **[%]**	o **[%]**
*0.1*	-	-	0.4	0.26	0.2	0.73	0.1	2.58
*0.2*	-	-	0.75	0.35	0.35	0.97	0.2	3.65
*0.2 S*	0.75	0.07	0.35	0.24	0.2	0.73	0.2	3.65
*0.5*	-	-	1.5	0.50	0.75	1.41	0.5	5.77
*0.5 S*	1.5	0.10	0.75	0.35	0.5	1.15	0.5	5.77
*1*	-	-	3	0.71	1.5	2.00	1	8.17
*3*	-	-	-	-	4.5	3.47	3	14.15

**Table 4:** Results for protective purpose LPCTs.

*AC*	$I_{n}$	
	ε [%]	o [%]
*5 TPE*	1	8.17
*5 P*	1	8.17
*10 P*	3	14.15

**Table 5:** Results for measuring purpose LPVTs.

*AC*	$0.8V_{n}$		$V_{n}$		$1.2V_{n}$	
	ε [%]	o [%]	ε [%]	o [%]	ε [%]	o [%]
*0.1*	0.1	2.07	0.1	2.58	0.1	3.10
*0.2*	0.2	2.92	0.2	3.65	0.2	4.38
*0.5*	0.5	4.62	0.5	5.77	0.5	6.93
*1*	1	6.53	1	8.17	1	9.80
*3*	3	11.32	3	14.15	3	16.97

**Table 6:** Results for protective purpose LPVTs.

*AC*	$0.01V_{n}$		$0.05V_{n}$		$0.2V_{n}$		$V_{n}$	
	ε [%]	o [%]	ε [%]	o [%]	ε [%]	o [%]	ε [%]	o [%]
*0.1 P*	0.5	0.12	0.2	0.73	0.1	2.07	0.1	2.58
*0.2 P*	1	0.16	0.4	1.03	0.2	2.92	0.2	3.65
*0.5 P*	2	0.23	1	1.63	0.5	4.62	0.5	5.77
*1 P*	4	0.33	2	2.31	1	6.53	1	8.17
*3 P*	6	0.40	3	2.83	3	11.32	3	14.15
*6 P*	12	0.57	6	4.00	6	16.01	6	20.01

**Table 7:** Electronic Instrument Transformer (EIT) features collected from off-the-shelf devices of different manufacturers.

DEVICE	FEATURE						
	Type	Rated Input	Rated Output	AC	Offset	Noise	Compliant down to
A	Voltage	700 V	10 V	0.2	±5 to ±13 mV	-	All
B	Voltage	4200 V	50 mA	1	±50 μA	10 μA	All
C	Voltage	2000 V	20 mA	0.3 *	±4 mA	16.5 μA	None
D	Voltage	1500 V	50 mA	1	±0.2 mA	-	All
E	Current	10 A	1.65 V	1	±5 mV	-	0.2$I_{n}$
F	Current	2000 A	10 V	0.016 *	±50 μV	-	All
G	Current	100 A	50 mA	0.5	±0.4 mA	-	0.2$I_{n}$
H	Current	25 A	25 mA	1	±0.7 mA	-	$I_{n}$

* these AC do not exist in [2], but the value refers to the indication provided by the manufacturer.
